# Synthesis and In Silico Study of Pectolinarigenin–Metronidazole Hybrid Molecule as Anti-*Helicobacter pylori*

**DOI:** 10.3390/molecules31122089

**Published:** 2026-06-14

**Authors:** Zeyneb Benramdane, Matteo Michelotti, Thamere Cheriet, Andrea Defant, Ines Mancini

**Affiliations:** 1 Laboratory of Bioorganic Chemistry, Department of Physics, University of Trento, 38123 Trento, Italy; zeyneb.benramdane@univ-msila.dz (Z.B.); matteo.michelotti03@gmail.com (M.M.); 2 Inorganic Materials Laboratory, Chemistry Department, Faculty of Sciences, University of M’Sila, Bordj Bou Arreridj road, M’Sila 28000, Algeria; 3 Unité de VALORISATION des Ressources Naturelles, Molécules Bioactives et Analyse Physicochimiques et Biologiques (VARENBIOMOL), Université des Frères Mentouri, Constantine 25017, Algeria; tamercheriet@umc.edu.dz; 4 Laboratoire de Génie Biologique, Valorisation et Innovation des Produits Agroalimentaires, Institut des Sciences Techniques et Appliquées-ISTA-Ain M’lila, Université Larbi Ben M’hidi, Oum El-Bouaghi 04000, Algeria; 5 Independent Researcher, 38121 Trento, Italy

**Keywords:** flavonoids, pectolinarigenin, metronidazole, molecular hybridization, antibacterial, *Helicobacter pylori* infection, ADME prediction, density functional theory (DFT) calculation, docking calculation, molecular dynamics simulation

## Abstract

Metronidazole is an antibiotic used to treat *Helicobacter pylori*, a bacterium responsible for chronic infections in humans that cause gastric inflammation, ulcers, and cancer. However, its long-term administration is limited by toxicity and increased resistance. In the search for more effective agents against *H. pylori* infection, molecular hybridization has now been applied to the synthesis of the new compound **3**. Its structure connects the metronidazole moiety to pectolinarigenin, the latter obtained by acid hydrolysis of glycosylated flavonoids isolated from the plant *Linaria reflexa* Desf. The NOE effect supported the C-7 functionalization of **3**, as evidenced by the energy-minimized DFT-calculated structure. The new molecule enriches the chemical space of known metronidazole–flavonoid analogs, among which the genistein derivative **2** was reported as the most active in inhibiting bacterial strains. The computational analysis of **2** and **3** compared with metronidazole as the reference has provided favorable data for both Absorption, Distribution, Metabolism, and Excretion (ADME) predictions and the probability of anti-*H. pylori* activity, besides rising docking evaluation on three specific targets and dynamics simulation as inhibitors of the flavodoxin enzyme. The results are promising for further in-depth biological investigation.

## 1. Introduction

*Helicobacter pylori* is a Gram-negative bacterium that infects up to 50% of the world’s human population and causes one of the most common chronic infections in humans. Related to socioeconomic status and hygiene conditions, it affects about 4.4 billion people worldwide, mostly in developing countries. It is a microaerophilic bacterium that requires low oxygen concentrations to survive in the stomach. The infection causes chronic progressive gastric inflammation, gastric and duodenal ulcers, and gastric cancer [1]. Anti-*H. pylori* treatment consists of broad-spectrum antibiotics in high doses (amoxicillin, clarithromycin, and metronidazole) and a proton pump inhibitor. However, long-term administration, due to recurring infections, can also cause serious side effects. Additionally, failure is mainly associated with antibiotic resistance, which is increasing globally. Therefore, *H. pylori* eradication treatment constitutes a relevant and urgent challenge, requiring the development of novel, efficient, and selective agents [2]. Furthermore, ongoing research on a vaccine against *H. pylori* infection has produced promising prototypes. However, there is no vaccine widely approved for clinical use yet.

Metronidazole is an antibiotic drug acting as a prodrug that is activated to produce the therapeutic agent by reduction reactions characteristic of anaerobic cell environments. These reactions reduce the nitro group, forming cytotoxic radicals that damage the DNA of target organisms, causing cell death [2]. Metronidazole remains a cornerstone antimicrobial agent extensively employed against anaerobic bacteria, protozoa, and parasites. However, the long-term administration causes serious adverse reactions and toxicity issues [3].

Flavonoids constitute a broad class of phytochemicals showing an extensive structural diversity. They are widespread in various plants, where they play roles in defense, and physiological and ecological processes [4]. In their role as constitutive antimicrobial barriers in plant kingdoms, these metabolites exhibit excellent activity against a wide range of pathogenic microorganisms through multiple mechanisms [5]. The pharmacological properties of flavonoids are well established, particularly for the treatment of chronic diseases and cancer [6]. One of the principal research interests is their bifunctional role in redox biology: as antioxidants under physiological conditions and, in some cases, as pro-oxidants that facilitate the generation of reactive oxygen species (ROS), which perturb cellular homeostasis and trigger apoptosis of tumor cells [7]. The favorable biocompatibility, low toxicity, and documented synergy with conventional antibiotics underscore flavonoids as attractive lead compounds for the development of new antimicrobial agents with enhanced efficacy and safety [8,9]. Pectolinarigenin is a flavonoid metabolite identified and isolated from many plants, and is the aglycone of linariin and pectolinarin. Pectolinarigenin has shown antibacterial, antioxidant, anti-inflammatory, and hepatoprotective activities [10].

In medicinal chemistry, molecular hybridization or conjugation is a valuable approach to optimize the study of new therapeutic agents for the treatment of cancer and a series of diseases. This strategy involves the covalent linkage between the pharmacophore units of two active compounds, or between two drugs themselves, also acting with different mechanisms of action, to improve the pharmacokinetic profile of the new chemical entity, its selectivity, and activity [11]. Molecular hybridization also enhances the aqueous solubility and intestinal absorption of an active drug, as well as extending its plasma half-life. Such structural modifications retain the biological activity of the original molecules and result in more favorable therapeutic profiles, associated with reduced toxicity [12]. The therapeutic efficacy of the hybrid molecule is generally higher than that of the single-target inhibitors.

**Figure 1:**
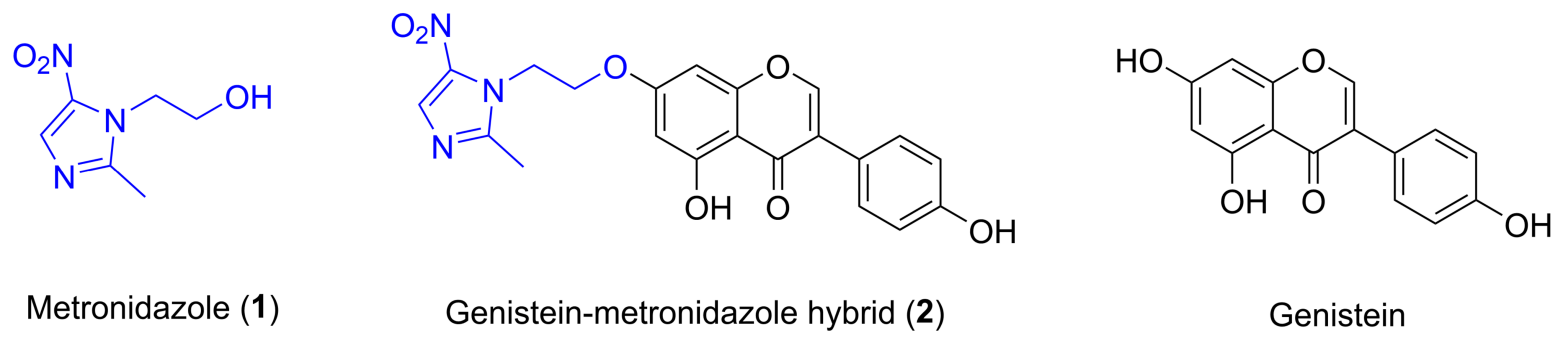
Molecular structure of the metronidazole–genistein **2** [14].

Metronidazole has been recognized as a privileged scaffold for the design of novel bioactive derivatives [13]. Known metronidazole conjugates have been reported. Regarding hybridization with flavonoids, the derivative formed by the chemical linkage of the natural genistein to the metronidazole moiety (**2** in Figure 1) was found to be the most active against *H. pylori* strains in a series of analogs. The obtained MIC value of 0.39 µg/mL was more than 50-fold higher than that of metronidazole against the ATCC 43504 strain and comparable to the antibiotic amoxicillin used as a control against four resistant clinical strains. Moreover, derivative **2** was nontoxic in a mouse model [14].

Urease is one of the main targets involved in *H. pylori* infection. Ureases are nickel-containing metalloenzymes of high molecular weight. They are present in bacteria where they can convert urea into ammonia and carbon dioxide, neutralizing the surrounding acidic environment. In this condition, the bacteria survive and can damage tissues, leading to gastritis, ulcers, and cancer. Targeting urease activity offers a promising strategy to reduce *H. pylori* pathogenicity and enhance its susceptibility to antibiotics. One potential way to eradicate *H. pylori* infection is the use of urease inhibitors as adjuvants, which exhibit an additive effect with current antibiotic treatment [15]. Flavonoid derivatives, also conjugated to metronidazole, have been studied as synthetic urease inhibitors, with structure–activity relationships investigated and molecular docking applied [16]. Flavodoxins are other novel, promising therapeutic targets against *H. pylori*. They are small soluble electron-transfer proteins absent in vertebrates but widely present in bacteria, where they participate in various metabolic pathways [17]. Additionally, another target can be identified in RdxA, an oxygen-insensitive NADPH nitroreductase that is the primary enzyme responsible for metronidazole activation in *H. pylori* [18]. Metronidazole resistance is mainly due to the inactivation of the oxidoreductase encoded by RdxA, with a proposed mechanism involving the reduction or abolition of electron carrier activity and the intracellular redox potential [19,20].

In this work, we focused on the synthesis of a pectolinarigenin–metronidazole hybrid molecule, and an in silico investigation was able to compare this new molecule with the most active metronidazole–flavonoid derivative **2**, of which only experimental data on the inhibition of *H. pylori* strain have been reported [14]. Based on known or potential target receptors involved in *H. pylori* infection [17,18,21], the comparison has included docking calculations and molecular dynamics simulation, supplemented by ADME-toxicity predictions for the two molecules.

## 2. Results and Discussion

### 2.1. Chemistry

**Scheme 1:**
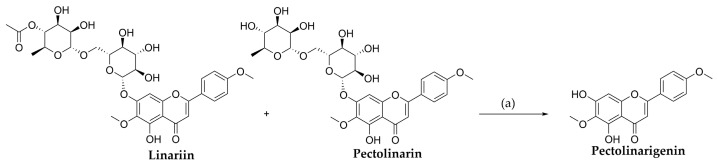
Production of pectolinarigenin. Reagent and condition: (a) 3M aq. HCl, MW, 100 °C, 1 h, 94%.

The genus *Linaria*, formerly placed in the family Scrophulariaceae, but now in Plantaginaceae, is known for its bioactive metabolites. The plant is used in folk medicine in African and Asian regions. A mixture of the glycosylated flavonoids linariin and pectolinarin was obtained from the methanol extract of the aerial parts of *Linaria reflexa* collected in Algeria [22]. Pectolinarigenin is a natural compound, but the sample used in this work was obtained as the common aglycone of linariin and pectolinarin via acid hydrolysis of their mixture under microwave irradiation, which replaced conventional heating, reducing the reaction time significantly (Scheme 1).

**Scheme 2:**
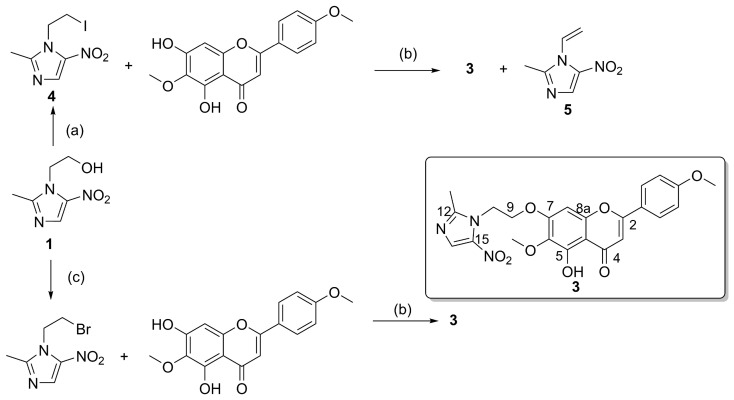
Synthesis of pectolinarigenin–metronidazole hybrid **3**. Reagents and conditions: (a) I_2_, Ph_3_P, imidazole, r.t., 44%; (b) K_2_CO_3_, acetone, reflux; (c) CBr_4_, Ph_3_P, THF, r.t., overnight, 82%. Arbitrary numbering is for convenience.

For the production of the desired **3**, we adopted the two-step sequence reported in Scheme 2. In the first step, the OH group of metronidazole was converted to iodide **4**, which was subsequently used in a nucleophilic substitution reaction with the anion of pectolinarigenin under basic conditions using an excess of potassium carbonate in acetone (Scheme 2). The refluxing condition in acetone, employed to facilitate the phenol deprotonation of pectolinarigenin, promoted the competitive elimination of iodide **4**, producing the terminal double bond of byproduct **5**. Therefore, this behavior prevented product **3** from achieving a good yield. We found an optimized method by using the brominated metronidazole derivative, replacing iodide **4**. In the S_N_2 reaction, the iodide is the best leaving group in the halide series. However, the competitive elimination to give byproduct **5** occurred under the conditions adopted. In contrast, the choice of bromide **6** minimized side reactions, thereby improving the yield of nucleophilic attack and providing the hybrid compound **3** in good yield and purity. Its purity was detected by the presence of a single chromatographic peak in HPLC analysis on reversed phase (Figure S1).

**Figure 2:**
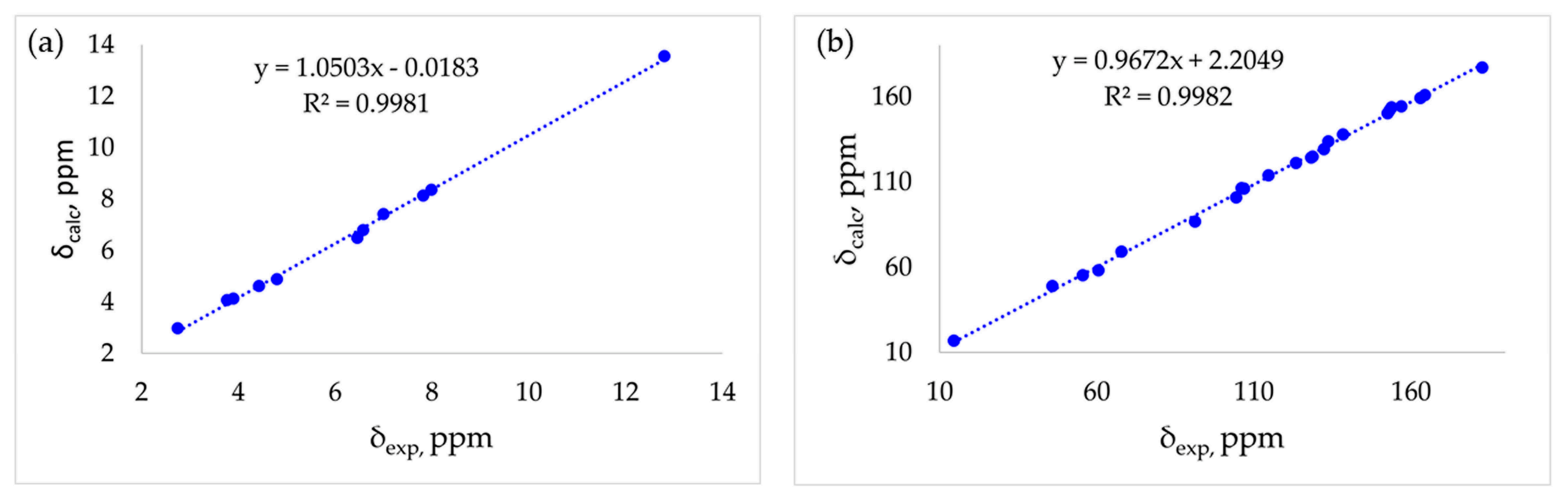
Correlation plots of calculated vs. experimental (**a**) ^1^H NMR and (**b**) ^13^C NMR chemical shifts in CDCl_3_ of **3**.

The structural characterization of **3** is based on high-resolution ESI-MS data and fragmentation experiments, the most significant of which is the one that generates the signal at *m*/*z* 313 from the [M − H]^−^ signal, attributable to the loss of the metronidazole moiety (Figure S2). The experimental ^1^H- and ^13^C NMR data (Figure S3) are fully assigned by comparison with density functional theory (DFT)-calculated chemical shifts, yielding mean absolute errors (MAEs) of 0.29 for ^1^H and 2.27 for ^13^C shifts (Table S1). Figure 2 shows a strong correlation between the experimental and calculated values.

**Figure 3:**
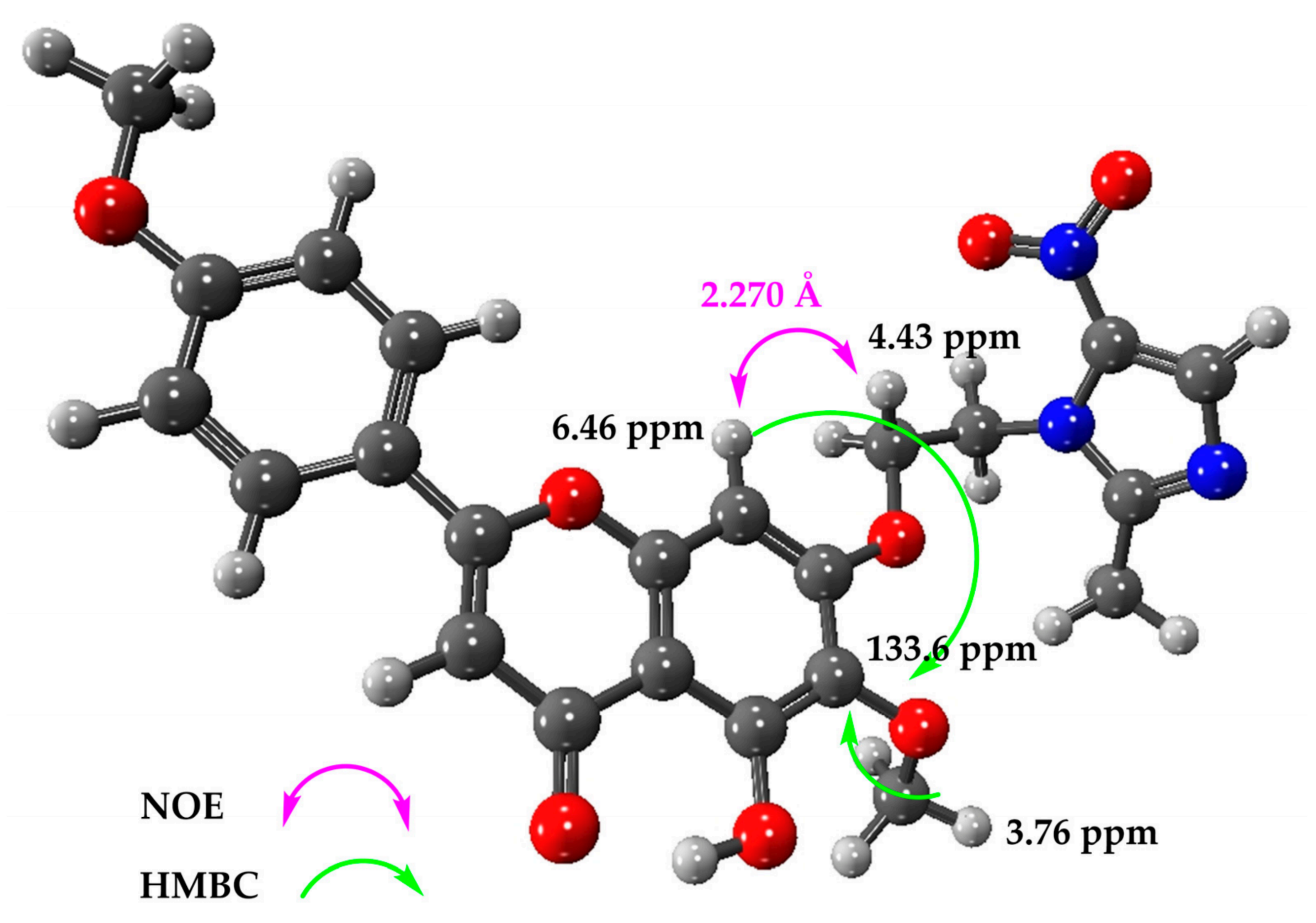
Energy-minimized structure obtained by DFT calculation in chloroform of **3**, reporting correlations from HMBC and NOESY experiments.

The functionalization of pectolinarigenin in C-7 with the metronidazole moiety was further established by a Heteronuclear Multiple Bond Correlation (HMBC) experiment (Figure S4) and the NOE effect observed in CDCl_3_ between H-8 at 6.46 ppm and H-9 at 4.43 ppm. This evidence is supported by the energy-minimized structure of **3** obtained by DFT calculation in chloroform, where the distance of 2.270 Å is compatible with the observed NOE (Figure 3).

### 2.2. In Silico Studies

The SwissADME virtual screening [23] of the pharmacokinetic parameters for the hybrid molecules **2** and **3**, compared with those of metronidazole, has yielded promising results. The same bioavailability value (0.55) was obtained for the three compounds (Table S2), as evident in the radar visualization, which accounts for a series of physicochemical properties (Figure S5). The comparable parameters of the two hybrid molecules are all within the bioavailability radar, although with values lower than those associated with metronidazole. In detail, the correlation between the molecular structures and these parameters shows that **2** and **3** have similar lipophilicity, higher than that of **1**, and moderate water solubility. Molecular flexibility is traceable to the number of rotatable bonds, which are more numerous in **2** and **3** than in **1**, and also to the smaller size of the metronidazole structure. The polarity, expressed as topological polar surface area (TPSA), is similar for **2** and **3** and is higher than that of metronidazole, but still acceptable for an antibiotic drug. The probability of activity against *H. pylori* has been evaluated using the Prediction of Activity Spectra for Substances (PASS) software(Way2Drug.com 2011-2026, Version 2), which predicts biological activity profiles of drug-like compounds using a structure–activity relationship (SAR) approach. The probability results reported in Table 1 highlight higher values for the hybrid molecules **2** and **3** than for the single structural components pectolinarigenin, genistein, and metronidazole, further indicating a synergistic effect of each flavonoid with metronidazole. Moreover, the high probability of **2** and **3** being active is associated with very low probabilities of being inactive (0.002 and 0.001, respectively).

Molecular docking of each compound as a ligand with the enzymes under investigation has provided the energy values reported in Table 2. The complexes involving the hybrid molecules **2** and **3** exhibit comparable values, indicating a greater stabilization compared with metronidazole (**1**).

**Figure 4:**
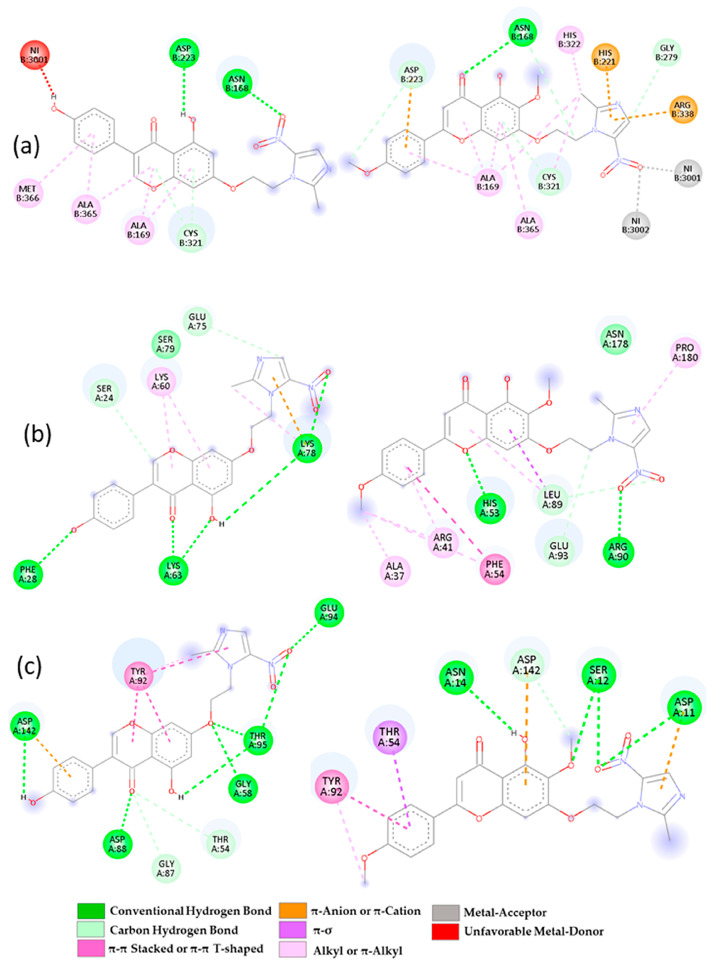
Two-dimensional representations for the interactions of **2** (left) and **3** (right) with *H. pylori* targets (**a**) urease (1E9Y), (**b**) RdxA oxygen-insensitive nitroreductase (3QDL), and (**c**) flavodoxin (2W5U).

Two-dimensional representations are displayed in Figure 4 for **2** and **3**, and Figures S6–S8 for metronidazole, genistein, and pectolinarigenin, respectively.

In detail, the complex of **3** with urease (1E9Y) is mainly involved in an H-bond with asparagine-168, also present in the **2**-complex, three π-ion interactions with aspartic-223, histidine-221 and arginine-338, and two interactions with both the nickel ions present in the active site. On the contrary, **2** shows an unfavorable interaction with one of these nickel ions. It is remarkable that in the case of urease, the interactions involving amino acids histidine-221, aspartic acid-223, alanine-365, and nickel ions 3001 and 3002 observed for ligand **3** are also present in the complex of acetohydroxamic acid detected by X-ray crystallography [24].

In the active site of RdxA oxygen-insensitive nitroreductase, the same interaction between arginine-90 and the nitro group in metronidazole and **3** is relevant for the bioactivation. Furthermore, additional stabilizing interactions are present with the flavonoid moiety in **3** and **2** (Figure 4).

In flavodoxin complexes, the main evidence is the different behavior of the ligands in the active site, as shown by tyrosine-92 interactions with the imidazole unit in **1** and **2**, and with the methoxy aryl moiety in **3** (Figure 4). The ligand **3** interacts with tyrosine-92, which is also involved in the complex with the original inhibitor [25].

**Figure 5:**
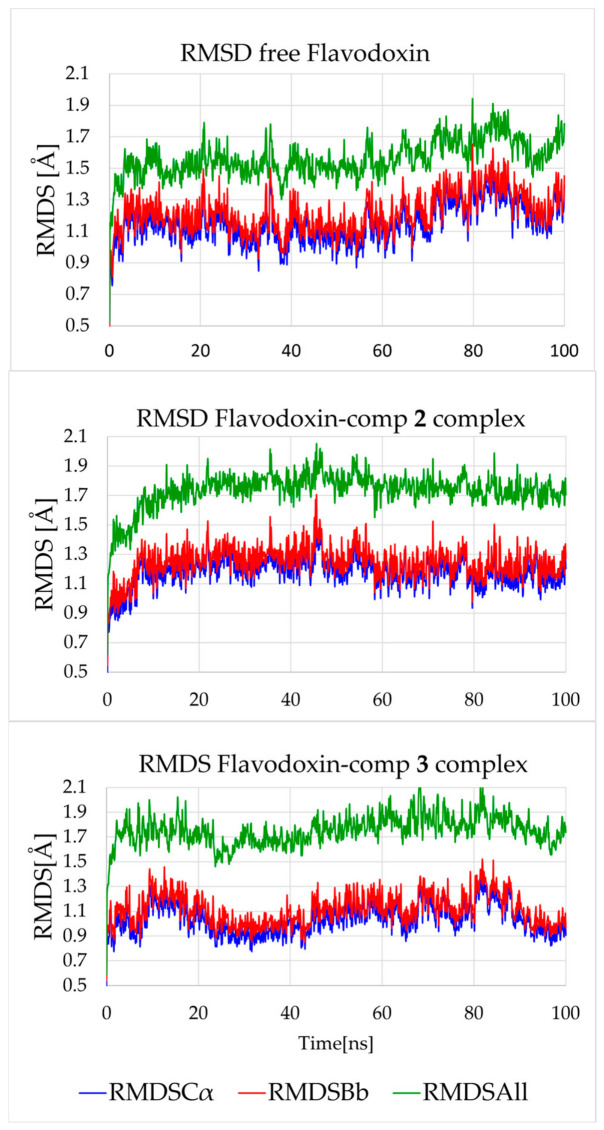
Root Mean Square Deviation (RMSD, in Å) from MD simulation during all simulation time for the indicated systems. Cα in blue, backbone in red, and all heavy atoms in green.

We performed molecular dynamics (MD) regarding flavodoxin because, among the enzymes under investigation in the previous docking calculation, it was the one accessible in complex with a suitable known inhibitor 5-amino-1-[2-nitro-4-(trifluoromethyl)phenyl]-1H-pyrazole-4-carbonitrile with an acceptable resolution (PDB-ID: 2W5U) [25]. The simulations for compounds **1**–**3** yielded a stable, similar profile of the total potential energy of the systems over time (Figure S9). The behavior obtained for each gyration radius was indicative of the protein’s compactness, which preserves the folding (Figure S10). In the evaluation of root mean square deviation, the flavodoxin-3 complex was comparable to the complex involving compound **2**, as evident from the profile for all heavy atoms in Figure 5.

**Figure 6:**
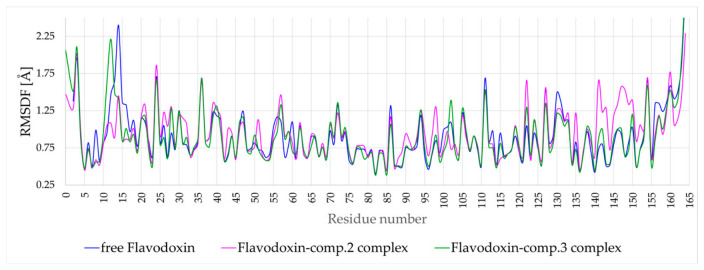
Root Mean Square Fluctuation (RMSF) from MD simulation during all simulation time for the indicated systems.

Regarding fluctuations of amino acid residues within the structure, Root Mean Square Fluctuation (RMSF) showed that the same amino acid residues were involved, with very similar ranges of motion for the free enzyme and its complex with **3**. On the contrary, the flavodoxin-2 complex shows a wider fluctuation in the region of amino acid residues 140–150 (Figure 6).

**Figure 7:**
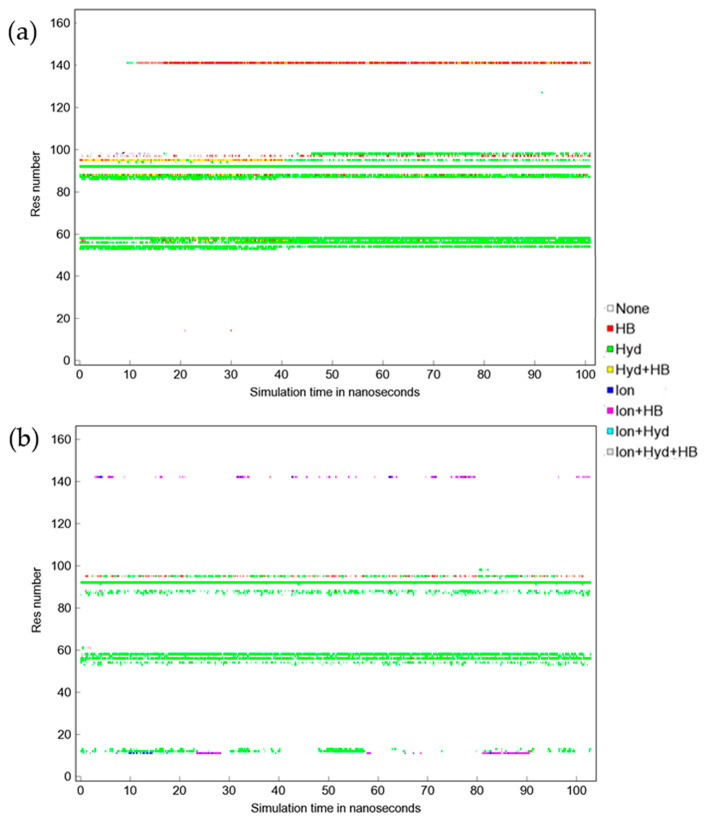
Per-residue contacts including interactions in function of the time involved for the hybrid molecules (**a**) **2**, and (**b**) **3**.

Additional MD data show the types of interactions by enzyme residues with each ligand **2** and **3** as a function of simulation time (Figure 7). The specific amino acid residues involved in the interactions are reported in Table 3. Eight residues are common in the two complexes (threonine-54, glycine-56, alanine-57, glycine-58, glycine-87, aspartic acid-88, tyrosine-92, and threonine-95), with tyrosine-92, which preserves the interactions throughout the simulation. Moreover, for **3**, the flavodoxin complex is stabilized by interactions with aspartic acid-11, serine-12, and glycine-13, not present in the complex with **2**.

MD simulation also allows the estimation of binding free energies between the ligand and enzyme using the molecular mechanics generalized born surface area (MM/GBSA) method. Comparable values were obtained for both the **2** and **3** complexes, corresponding to −20.5 and −19.0 kcal/mol, respectively.

**Figure 8:**
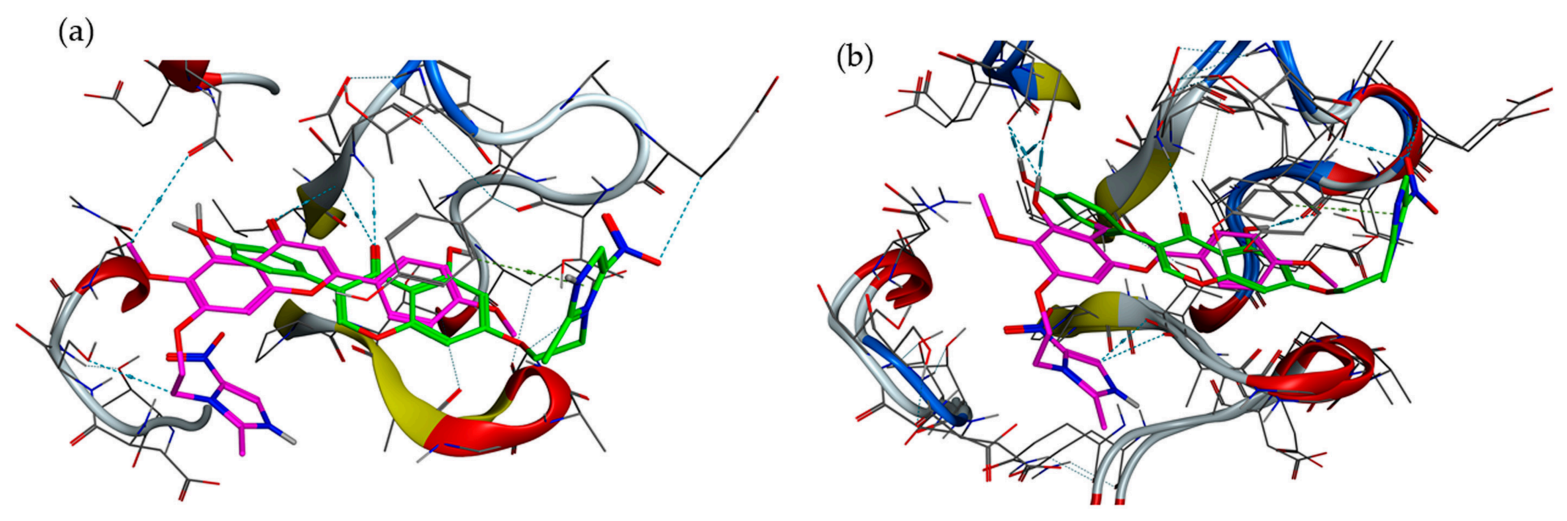
3D-views of **2** (in green) and **3** (in magenta) as ligands at the lowest energy inside the active site of flavodoxin by (**a**) docking calculation and (**b**) MD simulation.

In summary, Figure 8 displays a three-dimensional view of ligands **2** and **3** in the flavodoxin active site, as obtained by molecular docking and MD simulation. It is evident that the two molecules, both involved in stabilizing interactions, differ in position, with the metronidazole moiety pointing in opposite directions.

## 3. Materials and Methods

### 3.1. Chemistry

#### 3.1.1. Material and Instrumentation

All reagents and solvents were purchased from Sigma Aldrich and Merck (Avantor, Milan, Italy) and used without further purification. Microwave (MW) irradiation was preformed using a CEM microwave reactor (CEM srl, Bergamo, Italy), with input parameters entered in Synergy Discover software version 1.01. Reaction yields were calculated on the products after their chromatographic purification. Thin-layer chromatography (TLC) was performed using Merck Silica gel 60 F_254_ aluminum sheets 20 cm × 20 cm (VWR, Milan, Italy), under UV light at 254 nm for visualization, using Ce(SO_4_)_2_ as the developing agent upon heating. Preparative liquid chromatography (PLC) Merck Silica gel 60 F_254_, 0.5 mm glass plates (20 cm × 20 cm) (VWR, Milan, Italy) was used for purification. The purity of the product was confirmed by high-performance liquid chromatography on an Agilent 1200 HPLC system (Agilent Technologies, Waldbroon, Germany) using a Kinetex RP-18 100A column (250 × 4.60 mm, 5 µm particle size), (Phenomenex, Bologna, Italy) under the following conditions: flow 1 mL/min, injection 5 µL, temperature 30 °C, and UV-DAD detection at λ = 254, 280, and 310 nm. ^1^H and ^13^C NMR experiments were recorded on a Bruker-Avance 400 spectrometer (Bruker, Karlsruhe, Germany) using a 5 mm BBI probe, ^1^H at 400 MHz and ^13^C at 100 MHz, in CDCl_3_ (with values relative to TMS, δ_H_ 7.27 ppm and δ_C_ 77.00 ppm, respectively), or CD_3_OD (δ_H_ 3.31 ppm and δ_C_ 49.00 ppm). The chemical shift values were given in ppm and *J* values in Hz. The assignment of pectolinarigenin–metronidazole molecule was confirmed by long-range hetero-correlations obtained from a Heteronuclear Multiple Bond Correlation (HMBC) experiment. NMR data were analyzed using MestReNova software version 15.0. Electrospray ionization mass spectra (ESI-MS) were recorded using a Bruker Esquire-LC spectrometer (Bruker Daltonic, Karlsruhe, Germany) with an electrospray ion source used in positive or negative ion mode by direct infusion of a methanolic solution of the sample (source temperature 300 °C, drying gas N_2_ 4 L·min^−1^, and scan range *m*/*z* 100–1000). High-resolution ESI-MS measurements were obtained by direct infusion into an Orbitrap Fusion Tribrid mass spectrometer (Thermo Fisher Scientific, Waltham, MA, USA).

#### 3.1.2. Production and Structural Characterization of Pectolinarigenin

Pectolinarigenin was produced by semi-synthesis starting from a natural mixture of linariin/pectolinarin. The dried aerial parts of *Linaria reflexa* Desf. collected in Algeria (70.7 g) were cut into small pieces and subjected to maceration at room temperature in MeOH/H_2_O (80:20 *v*/*v*) for 72 h (×4) as reported in the literature [22]. After filtration, the extraction solution was dried under reduced pressure (40 °C) to obtain dry crude extract (26.65 g). This extract was diluted with distilled water (500 mL) and subjected to successive extractions using petroleum ether (300 mL), chloroform (CHCl_3_, 300 mL × 3), and ethyl acetate (EtOAc, 300 mL × 6). The dried EtOAc phase was treated with MeOH to afford a light yellow precipitate, which was washed several times with the same solvent and then dried under reduced pressure (586.4 mg). A 2:1 mixture of linariin/pectolinarin was identified by NMR and MS analyses, consistent with reported data [22] (Scheme 1).

The linariin/pectolinarin mixture (277.2 mg) and 3M aq. HCl (25 mL) was refluxed at 100 °C under vigorous stirring for 3 h, then stirred overnight at room temperature. The reaction progress was monitored by TLC (silica gel, using dichloromethane and acetone 9:1 as eluent). The combined organic phases from dichloromethane extracts (15 mL × 3) were washed twice with distilled water, dried over anhydrous sodium sulfate, and concentrated under reduced pressure. The obtained powder (125.8 mg, 94.0%) was identified as pectolinarigenin. Replacing conventional heating with microwave irradiation (100 W, 100 °C, 1 h) provided acid hydrolysis with a similar yield.

Data of pectolinarigenin (=5,7-Dihydroxy-6-methoxy-2-(4-methoxyphenyl)-4H-1-benzopyran-4-one). Pale yellow powder. HPLC analysis: RP-18, acetonitrile/water 80:20, t_R_: 5.9 min, purity 98.1%. ^1^H NMR (400 MHz, CDCl_3_): δ 13.10 (s, 1H), 7.84 (d, *J* = 9.0 Hz, 2H), 7.02 (d, *J* = 8.8 Hz, 2H), 6.59 (s, 1H), 6.57 (s, 1H), 4.05 (s, 3H), 3.90 (s, 3H). ESI(+)-MS: *m*/*z* 315.1 [M + H]^+^, 337.3 [M + Na]^+^, 650.9 [2M + Na]^+^, ESI(−)-MS: *m*/*z* 313.0 [M − H]^−^.

#### 3.1.3. Synthesis and Structural Characterization of Metronidazole-Pectolinarigenin **3**

The Sequence is Depicted in Scheme 2.

##### 1-(2-Iodoethyl)-2-methyl-5-nitro-1H-imidazole (**4**)

Metronidazole (52.8 mg, 0.309 mmol), molecular iodine (152.2 mg, 0.5997 mmol), imidazole (62.2 mg), and triphenylphosphine (150.7 mg) were mixed in 10 mL of THF. After the reaction was complete (monitored by TLC using 9:1 dichloromethane–acetone as the eluent), the crude mixture was diluted with water and extracted with dichloromethane (10 mL × 3). The combined organic layers were dried over anhydrous Na_2_SO_4_, filtered, and concentrated under reduced pressure. PLC under the same conditions used for TLC allowed the isolation of **4** as a yellow powder, collecting the band dissolved in dichloromethane and ethanol, concentrating to dryness, and repeating the procedure to remove the triphenylphosphine oxide byproduct (38.1 mg, 44%).

Data. ^1^H NMR (400 MHz, CDCl_3_): 7.95 (s, 1H), 5.84 (d, *J* = 6.2 Hz, 2H), 4.83 (d, *J* = 16.3 Hz, 2H), 4.43 (d, *J* = 8.4 Hz, 1H), 2.51 (s, 3H) [14].

##### 1-(2-Bromoethyl)-2-methyl-5-nitro-1H-imidazole (**6**)

Metronidazole (105.7 mg, 0.6176 mmol) and carbon tetrabromide (257.0 mg, 0.7750 mmol) were dissolved in anhydrous tetrahydrofuran (THF, 10 mL) and cooled to 0 °C. Then, triphenylphosphine (190.6 mg) was added slowly while stirring. After 15 min, the reaction mixture was warmed to room temperature and stirred overnight. The reaction was monitored by TLC (silica gel, dichloromethane/acetone 9:1). The crude mixture was diluted with water and extracted with dichloromethane (10 mL × 3). The combined organic layers were dried over anhydrous sodium sulfate, filtered, and concentrated under reduced pressure. Purification was carried out by PLC using the same eluent employed in TLC analysis. Product **6** was obtained as a pale yellow powder from the acetone-extracted band, which was removed by evaporation (118.3 mg, 82%).

Data. ^1^H NMR (400 MHz, CDCl_3_) δ 7.99 (s, 1H), 4.69 (t, *J* = 6.2 Hz, 2H), 3.71 (t, *J* = 6.2 Hz, 2H), 2.58 (s, 3H) [26].

##### 5-Hydroxy-6-methoxy-2-(4-methoxyphenyl)-7-(2-(2-methyl-5-nitro-1H-imidazol-1-yl)ethoxy)-4H-chromen-4-one (**3**)

Method A. A mixture of pectolinarigenin (13.8 mg, 0.0439 mmol) and potassium carbonate (14.1 mg, 0.102 mmol) in acetone (1.0 mL) was refluxed for 1 h. Then, a solution of iodide **4** (Scheme 2, 12.6 mg, 0.0448 mmol) in acetone (0.5 mL) was added, obtaining a heterogeneous mixture that was stirred under reflux conditions for an additional 72 h. The progress of the reaction was monitored by TLC using dichloromethane/acetone (9:1) as the eluent. Once the iodide had reacted to completion, the mixture was filtered and evaporated to dryness under reduced pressure. The desired product **3** was recovered by PLC purification of the crude mixture using the same TLC eluent (1.2 mg, 6%). However, the major product was the byproduct **5**, corresponding to the less polar band.

Data of 2-methyl-5-nitro-1-vinyl-1*H*-imidazole (**5**). ^1^H NMR (400 MHz, CDCl_3_): 7.95 (s, 1H), 5.84 (d, *J* = 6.2 Hz, 2H), 4.83 (d, *J* = 16.3 Hz, 2H), 4.43 (d, *J* = 8.4 Hz, 1H), 2.51 (s, 3H) [27].

Method B. The reaction and the subsequent chromatographic purification were carried out under the same conditions as described in method A, but using iodide **4** replaced by bromide **6**. A higher amount of pure product **3** (13.8 mg, 66%) and a negligible amount of byproduct **5** were obtained by the same workup described for method A. The product was recovered by PLC as a glassy yellow solid. It displayed a 98.4% purity by reversed phase HPLC analysis (RP-18, acetonitrile/water 50:50, t_R_: 3.3 min), (Figure S1).

Data of 5-hydroxy-6-methoxy-2-(4-methoxyphenyl)-7-(2-(2-methyl-5-nitro-1*H*-imidazol-1-yl)ethoxy)-4*H*-chromen-4-one (**3**). ^1^H NMR (400 MHz, CDCl_3_, assignments in Table S1) δ 12.80 (s, 1H), 7.99 (s, 1H), 7.82 (d, *J* = 8.7 Hz, 2H), 7.00 (d, *J* = 8.7 Hz, 2H), 6.58 (s, 1H), 6.46 (s, 1H), 4.80 (t, *J* = 4.7 Hz, 2H), 4.43 (t, *J* = 4.7 Hz, 2H), 3.89 (s, 3H), 3.76 (s, 3H), 2.74 (s, 3H). ^13^C NMR (100 MHz, CDCl_3_, assignments in Table S1) δ 182.7, 164.4, 162.9, 156.9, 153.7, 153.1, 152.6, 138.3, 133.6, 132.3, 128.7, 128.2, 123.4, 114.6, 106.0, 104.3, 91.2, 67.8, 60.6, 55.6, 45.9, 14.5. ESI(+)-MS *m*/*z*: 490.2 [M + Na]^+^, MS/MS (490): *m*/*z* 488, 475, 429; ESI(−)-MS: *m*/*z* 466.3 [M − H]^−^, MS/MS (466): *m*/*z* 436, 422, 313 (Figure S2). HR-ESIMS: *m*/*z* 468.13929 [M + H]^+^, calcd. for C_23_H_22_N_3_O_8_: 468.140141; *m*/*z* 490.12132 [M + Na]^+^, calcd. for C_23_H_21_N_3_ NaO_8_: 490.12209.

### 3.2. Computational Analyses

#### 3.2.1. Minimized Structure of Compound **3** by DFT Calculation

The structure of **3** was built in MOE, and a conformational analysis was performed using the LowModeMD method with default parameters. Due to the large number of possible conformers, it was decided as a first approximation to focus on the conformer with the lowest relative energy. The conformer with the lowest energy was optimized in restricted mode both in vacuo and in implicit solvent via the Conductor-like Polarized Continuum Model (C-PCM) [28], (chloroform), using the Gaussian 16W revision A.03 program set [29]. The adopted basis set was 6-31+G(d,p), and the gradient-corrected DFT with the three-parameter hybrid functional (B3) [30] for the exchange part and the Lee–Yang–Parr correlation function [31] were selected. For the NMR calculation, the gauge-including atomic orbital (GIAO) [32] was employed using the basis set aug-cc-pVDZ in combination with the meta-generalized gradient approximation (M-GGA) M06-L functional [33,34] for ^1^H calculation and the same basis set in combination with the M-GGA TPSSh functional [35] for ^13^C. The chemical shift (δ) value was obtained from the equation δi = σ_TMS_ − σi, where σi is the isotropic shift constant for each nucleus, and σ_TMS_ is related to the reference tetramethylsilane (TMS), calculated at the same level of theory.

#### 3.2.2. ADME Prediction

ADME prediction was performed using the Swiss-ADME online server [36]. PASS software(Way2Drug.com 2011-2026, Version 2) was used to estimate biological activity [37].

#### 3.2.3. Molecular Docking Procedure

The ligand structures were built in MOE, minimized using the MMFF94-x force field, and saved in pdb format. AutoDock Tools (ADT) package version 1.5.7 [38] was employed to generate the docking input files, whereas AutoDock Vina 1.1.2 [39] was used for docking calculations. The structures of *Helicobacter pylori* urease complexed with acetohydroxamic acid (PDB ID: 1E9Y) [24], *H. pylori* flavodoxin in complex with 5-amino-1-[2-nitro-4-(trifluoromethyl)phenyl]-1H-pyrazole-4-carbonitrile (PDB ID: 2W5U) [25], and *H. pylori* RdxA oxygen-insensitive nitroreductase containing the flavin mononucleotide (PDB ID: 3QDL) [40] were determined by X-ray crystallography with a resolution of 3.0 Å, 2.6 Å, and 2.0 Å respectively. The structures were prepared according to the standard method, described by Vigna et al. [41].

A grid box of 16 × 16 × 16 Å in x, y, z coordinates, spacing of 1.00 Å and centered at x = 128, y = 129, z = 87 for 1EY9, a grid box of 18 × 18 × 18 Å in x, y, z coordinates, spacing of 1.00 Å and centered at x = 20.8, y = −0.14, z = 30 for 2W5U, and a grid box of 60 × 50 × 56 Å in x, y, z coordinates, spacing of 1.00 Å and centered at x = 17, y = 1.1, z = 0.07 for 3QDL were created. Vina parameters were set as follows: exhaustiveness of the local search = 100 and number of conformations to calculate = 10. Results are expressed as energy associated with each ligand–enzyme complex in terms of Gibbs free energy values. The visual inspection of the ligand–enzyme interactions was displayed using the Biovia Discovery Studio visualizer (Discovery Studio Visualizer v25.1.0.24284) [42].

#### 3.2.4. Molecular Dynamics Procedure

Calculations were carried out using Yasara software version 26.5.5 (YASARA Biosciences GmBH, Vienna, Austria), adopting the standard macro [43]. The structures of *H. pylori* flavodoxin in complex with **2** or **3** were obtained from molecular docking calculations and saved in pdb file format. Each pdb file was cleaned, hydrogen atoms were added, and the hydrogen-bond network was optimized. The simulation was performed for 100 ns, and the trajectories were saved every 100 ps [44,45]. The intermolecular force calculation was saved every two simulation sub-steps, whereas simulation snapshots were taken every 100 ps. The binding free energy of each ligand was calculated through the MM/GBSA method [46].

The system composition for **2** simulation resulted in 2413 atoms for the protein, 48 atoms in the ligand, 30 Na^+^ ions, 18 Cl^−^ ions, and 8131 water molecules, for a total of 26,902 atoms in the soup. The system composition for **3** simulation resulted in 2413 atoms for the protein, 56 atoms in ligand **3**, 29 Na^+^ ions, 18 Cl^−^ ions, and 8085 water molecules, for a total of 26,771 atoms in the soup.

## 4. Conclusions

The synthesis of a molecule structurally derived from pectolinarigenin and the antibiotic drug metronidazole has been optimized, starting from a metronidazole bromo-derivative and pectolinarigenin, which was accessible via microwave-assisted acid hydrolysis of a mixture of glycosylated flavonoids isolated from the plant *Linaria reflexa* Desf. The comparison of this new hybrid molecule with that derived from genistein and metronidazole, reported in the literature as the most active of a series of analogs tested against some *H. pylori* strains, has included the prediction of chemico-physical parameters to evaluate bioavailability, docking calculation considering three enzymes as potential *H. pylori* targets, and MD simulation for the complexes with flavodoxin enzyme. In summary, the comparable computational behavior of the two hybrid molecules suggests that these structures may be promising candidates for addressing the resistance to metronidazole as an anti-*H. pylori* antibiotic. The next biological evaluation addressed by these in silico studies should focus on the assays of activity against bacterial strains and on enzyme inhibition by the two hybrid molecules.

## Figures and Tables

**Table 1:** Anti-*Helicobecter pylori* activity predicted by PASS online server.

Compound	Anti-*H. pylori* Activity
Metronidazole (**1**)	0.679
**2**	0.836
**3**	0.844
Pectolinarigenin	0.521
Genistein	0.567

**Table 2:** Binding energy score (in kcal/mol) by Vina docking calculation of the indicated compounds as ligands with the *H. pylori* targets urease (1E9Y), flavodoxin (2W5U), and RdxA oxygen-insensitive nitroreductase (3QDL).

Compound	Binding Energy (kcal/mol)		
	1E9Y	2W5U	3QDL
Metronidazole (**1**)	−5.4	−5.2	−4.6
**2**	−7.0	−8.5	−6.9
**3**	−8.0	−8.2	−7.4
Pectolinarigenin	−6.7	−7.6	−7.6
Genistein	−7.0	−7.9	−6.5

**Table 3:** MD data on the types of interaction (HB, Hydrogen bond; Hyd, hydrophobic; Ion, ionic) with the indicated aminoacidic residues and the percentage of involvement during all simulation time.

Compound **2**					
Residue	HB	Hyd	Hyd + HB	Ion	%Tot
PRO-53		27			27
THR-54		80			80
GLY-56		80			80
ALA-57		45	5		50
GLY-58		90	6		96
LEU-86		21			21
GLY-87		84			84
ASP-88	12	48	20		80
TYR-92		100			100
THR-95	7	30	32		69
ALA-97	26				26
GLU-141	68	2	15		85
Compound **3**					
Residue	HB	Hyd	Ion	Ion + Hyd + HB	%Tot
ASP-11			12		12
SER-12		29			29
GLY-13		12			12
THR-54		25			25
GLY-56		80			80
ALA-57		16			16
GLY-58		84			84
GLY-87		24			24
ASP-88	1	27	2	1	31
TYR-92		100			100
THR-95	16	29			45
ASP-142	1		12		13

## Data Availability

The original contributions presented in this study are included in the article/Supplementary Materials. Further inquiries can be directed to the corresponding authors.

## Supplementary Materials

The following supporting information can be downloaded at: https://www.mdpi.com/article/10.3390/molecules31122089/s1, Figure S1: HPLC analysis of **3**; Figure S2: ESIMS spectra of **3**; Figure S3: NMR spectra of **3**; Table S1: Experimental and calculated chemical shifts of **3**; Figure S4: HMBC spectrum of **3**; Figure S5: Bioavailability radar by SwissADME tool of compounds **1**–**3**; Table S2: ADME prediction for **1**–**3** by SwissADME; Figures S6–S8: 2D views of interactions for metronidazole, pectolinarigenin and genistein with the three enzymes; Figures S9 and S10: Data from MD simulation.

## Abbreviations

The following abbreviations are used in this manuscript:

ADME	Absorption, Distribution, Metabolism, and Excretion
DFT	Density Functional Theory
ESI-MS	ElectroSpray Mass Spectrometry
HMBC	Heteronuclear Multiple Bond Correlation
HPLC	High-Performance Liquid Chromatography
MM/GBSA	Molecular Mechanics/Generalized Born Surface Area
NMR	Nuclear Magnetic Resonance
NOESY	Nuclear Overhauser Effect Spectroscopy
PASS	Prediction of Activity Spectra for Substances
PDB	Protein Data Bank
SAR	Structure–Activity Relationship
