# Supplementary material for: Synthesis and In Silico Study of Pectolinarigenin–Metronidazole Hybrid Molecule as Anti-Helicobacter pylori
Source: Molecules. 2026 Jun 14;31(12):2089. doi: 10.3390/molecules31122089 (PMC13304794; doi:10.3390/molecules31122089)
Supplement: Supplementary file 1 [file molecules-31-02089-s001.zip › molecules-4328149-supplementary.pdf]

Supplementary Materials

Synthesis and *In Silico* Study as Anti-*Helicobacter pylori* of Pectolinarigenin-Metronidazole Hybrid Molecule

Zeyneb Benramdane, Matteo Michelotti, Thamere Cheriet, Andrea Defant* and Ines Mancini *

Table of Contents

Table S1. Experimental and calculated chemical shifts for metronidazole-pectolinarigenin compound **3**. Arbitrary numbering of atoms as reported on the structure in Scheme 2. MAE = Mean absolute error.

Figure S1. HPLC analysis of **3** (PhenoMenex Kinetex RP-18 column acetonitrile/water 80:20, flow 1 mL/min, λ = 254): t_R: 3.3 min, purity 98.4 %.

Figure S2. MS analysis of **3**: enlarged area of the high resolution ESIMS experiment on $[M+H]^+$ signal, and fragmentation experiments ESI(+)-MS/MS on *m/z* 490 and ESI(−)MS/MS on *m/z* 466**.**

Figure S3. ^{1}H-NMR (400MHz) and ^{13}CNMR (100MHz) spectra of **3** in $CDCl_3$.

Figure S4. Enlarged area of the Heteronuclear Multiple Bond Correlation (HMBC) spectrum of **3** in $CDCl_3$.

Figure S5. Bioavailability Radar by SwissADME tool of metronidazole (**1**) and hybrid molecules **2** and **3**. The six physicochemical properties taken into account are: lipophilicity (LIPO), size, polarity (POLAR), solubility (INSOLU), saturation (INSATU), and flexibility (FLEX).

Table S2. ADME Prediction of the most significant parameters for **1-3** by SwissADME online server.

Figure S6. Two-dimensional representations for the interactions of metronidazole (**1**) with *H. pylori* targets (a) urease (1E9Y), (b) RdxA oxygen-insensitive nitroreductase (3QDL), and (c) flavodoxin (2W5U).

Figure S7. Two-dimensional representations for the interactions of pectolinarigenin with *H. pylori* targets (a) urease (1E9Y), (b) RdxA oxygen-insensitive nitroreductase (3QDL), and (c) flavodoxin (2W5U).

Figure S8. Two-dimensional representations for the interactions of genistein with *H. pylori* targets (a) urease (1E9Y), (b) RdxA oxygen-insensitive nitroreductase (3QDL), and (c) flavodoxin (2W5U).

Figure S9. Data from MD simulation: total potential energy of the indicated systems during all simulation time

Figure S10. Data from MD simulation: radius of gyration (in Å) during all simulation time for the indicated systems.

Table S1. Experimental and calculated chemical shifts for metronidazole-pectolinarigenin compound **3**. Arbitrary numbering of atoms as reported on the structure in Scheme 2. MAE = Mean absolute error.

**Position**	**Exptl.**	**Calcd.**	**|Δ|**
OH	12.80	13.56	0.76
H-14	7.99	8.36	0.37
H-2'/H-6'	7.82	8.14	0.32
H-3'/H-5'	7.00	7.43	0.43
H-3	6.58	6.79	0.21
H-8	6.46	6.50	0.04
2H-10	4.80	4.90	0.10
2H-9	4.43	4.63	0.20
OCH_3	3.89	4.13	0.24
OCH_3	3.76	4.08	0.32
CH_3	2.74	2.98	0.24
**MAE**			**0.29**

**Position**	**Exptl.**	**Calcd.**	**|Δ|**
C-4	182.7	177	5.7
C-2	164.4	160.8	3.6
C-4'	162.9	159.1	3.8
C-12	156.9	154.3	2.6
C-7	153.7	153.8	0.1
C-5	153.1	152.0	1.1
C-8a	152.6	150.2	2.4
C-15	138.3	137.9	0.4
C-3	133.6	133.8	0.2
C-14	132.3	129.1	3.2
C-2' or C-6'	128.7	124.9	3.8
C-6' or C-2'	128.2	124.2	4.0
C-1'	123.4	121.1	2.3
C-3' or C-5'	114.6	114.0	0.6
C-5' or C-3'	106.0	106.4	0.4
C-4a	106.9	106.1	0.8
C-3	104.3	101.0	3.3
C-8	91.2	86.7	4.5
C-9	67.8	69.2	1.4
OCH_3	60.6	58.3	2.3
OCH_3	55.6	55.4	0.2
C-10	45.9	48.9	3
CH_3	14.5	17.0	2.5
**MAE**			**2.27**

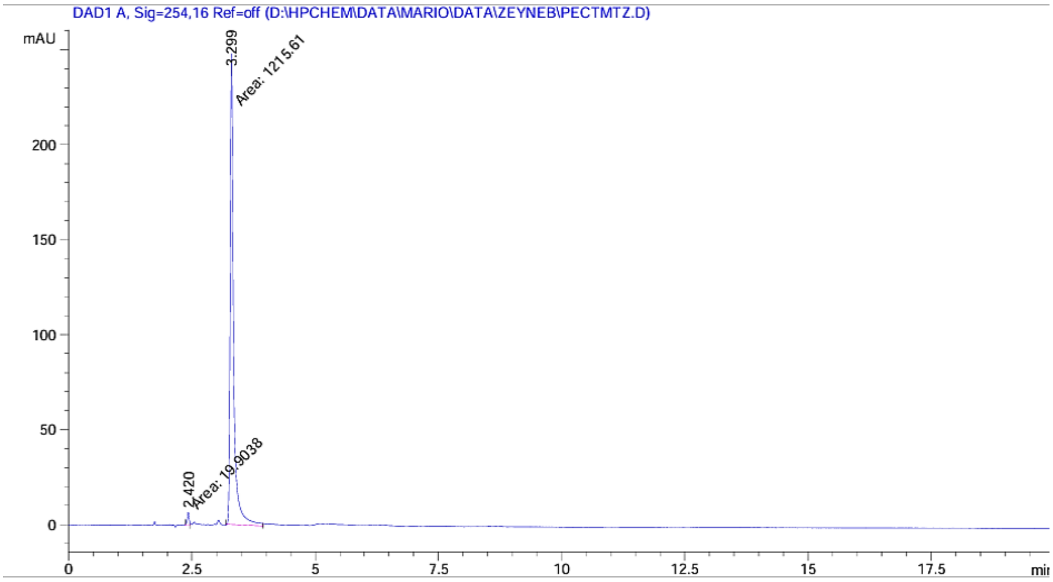

Figure S1. HPLC analysis of **3** (PhenoMenex Kinetex RP-18 column, acetonitrile/water 80:20, flow 1 mL/min, λ = 254): t_R: 3.3 min, purity 98.4 (%).

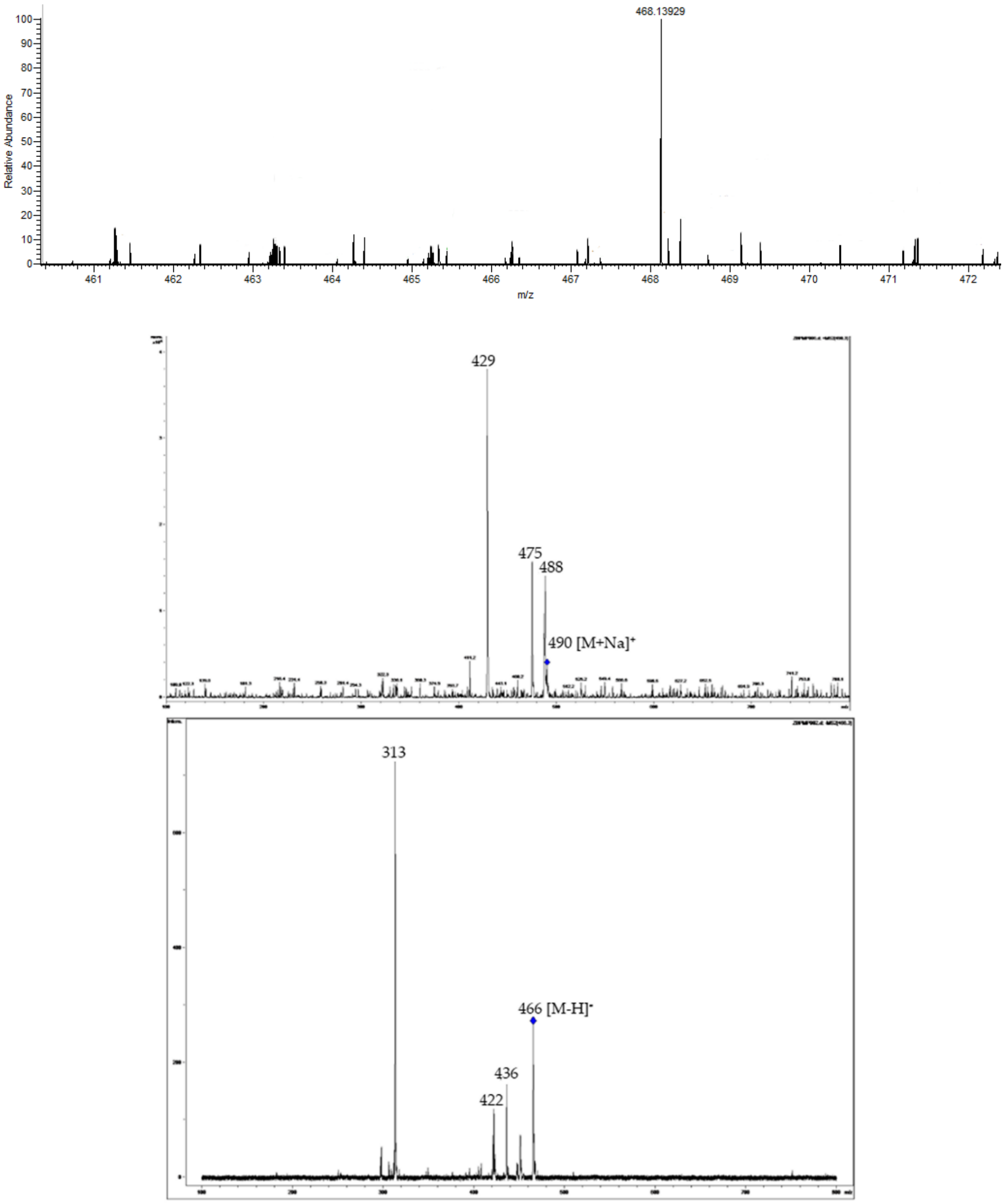

Figure S2. MS analysis of **3**: enlarged area of the high resolution ESIMS experiment on $[M+H]^+$ signal (top), and fragmentation experiments ESI(+)-MS/MS on *m*/*z* 490 (middle) and ESI(−)MS/MS on *m*/*z* 466 (down).

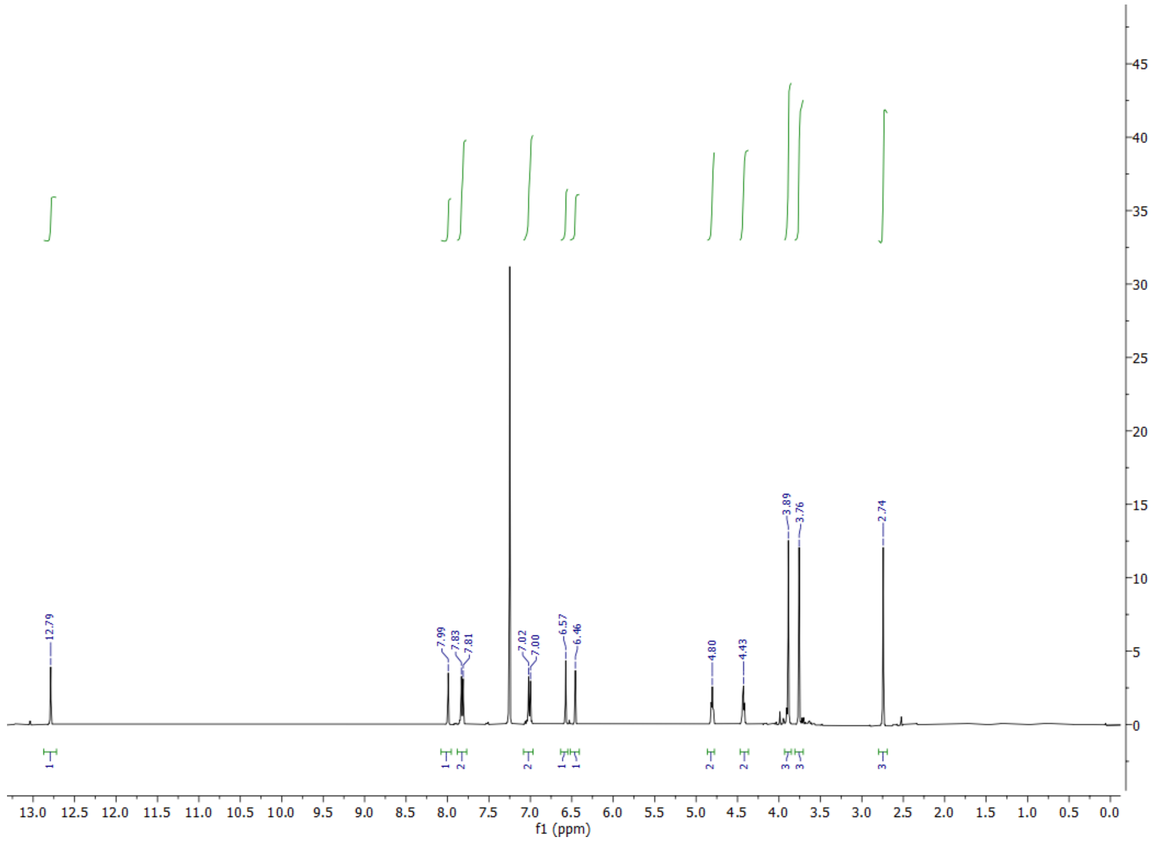

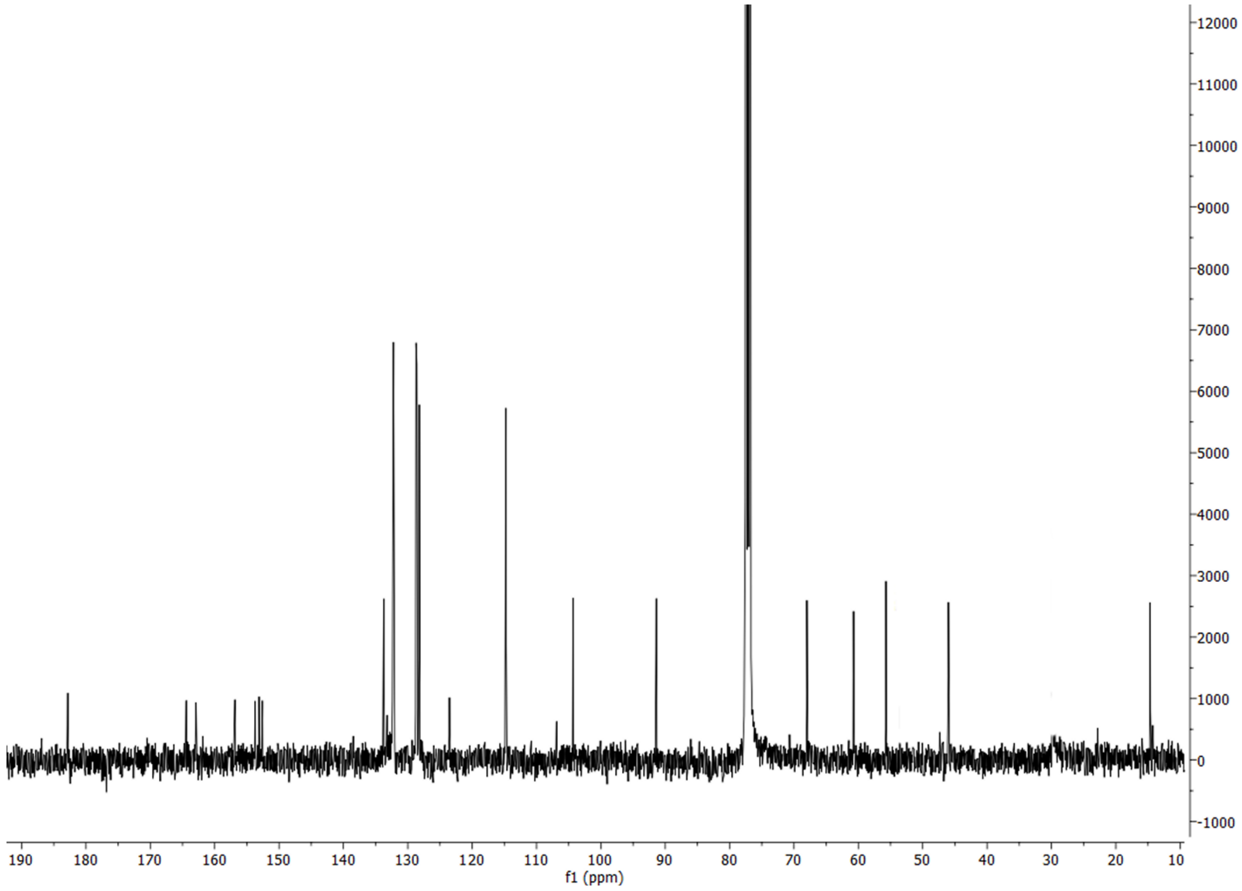

Figure S3. ^{1}H-NMR (400 MHz) and ^{13}CNMR (100 MHz) spectra of **3** in $CDCl_3$

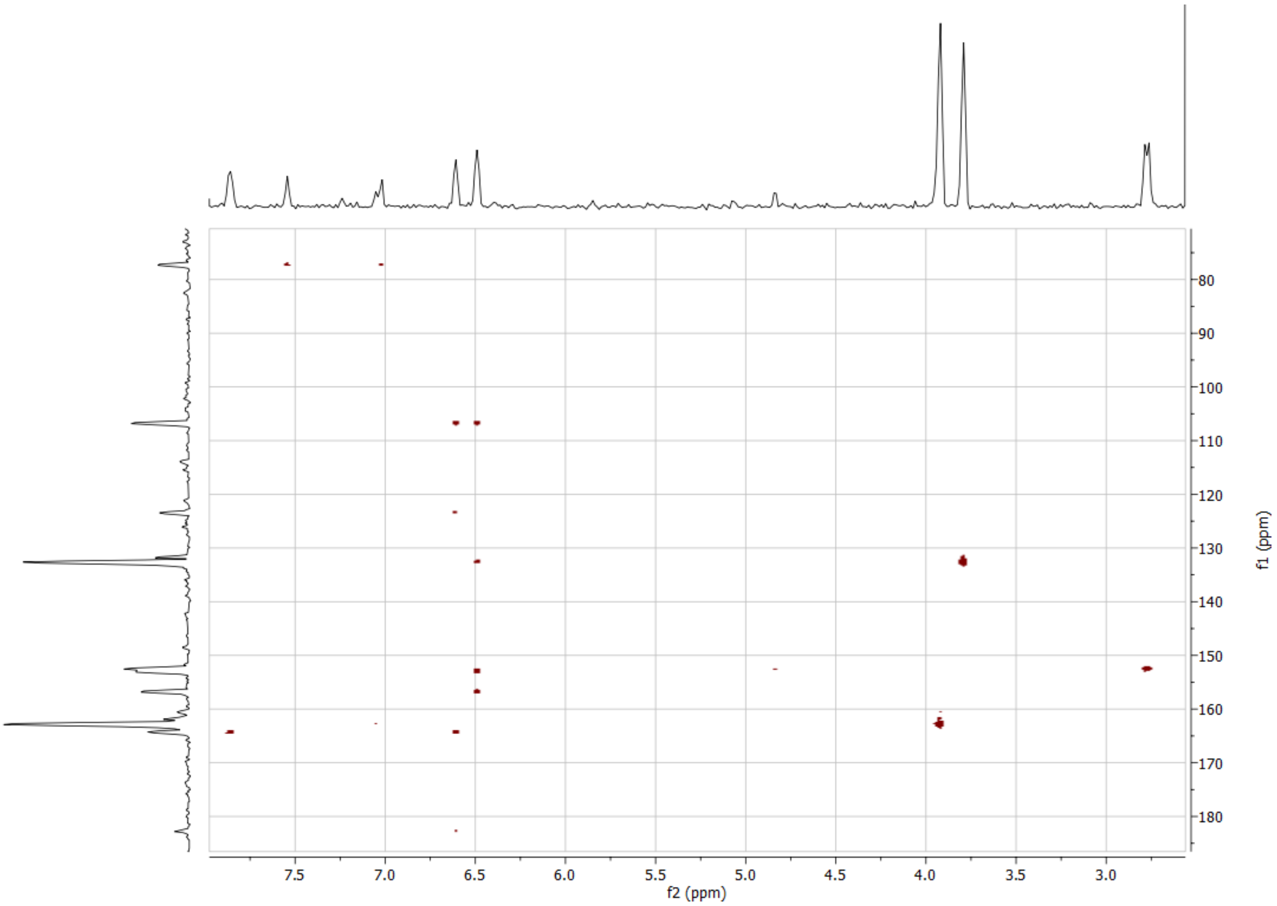

Figure S4. Enlarged area of the Heteronuclear Multiple Bond Correlation (HMBC) spectrum of **3** in $CDCl_3$.

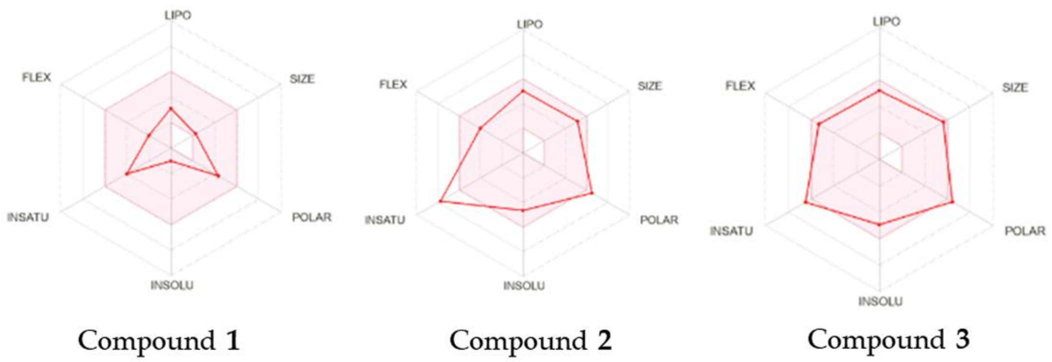

Figure S5. Bioavailability Radar by SwissADME tool of metronidazole (**1**) and hybrid molecules **2** and **3**. The six physicochemical properties taken into account are: lipophilicity (LIPO), size, polarity (POLAR), solubility (INSOLU), saturation (INSATU), and flexibility (FLEX).

Table S2. ADME Prediction of the most significant parameters for **1-3** by SwissADME online server

Parameters	Compound **1**	Compound **2**	Compound **3**
Number of rotatable bonds	3	6	8
Number of H-bond acceptors	4	8	9
Number of H-bond donors	1	2	1
TPSA (Å^2)	83.87	143.54	141.77
Consensus Log P	-0.23	2.11	2.47
ESOL Solubility Class	Very soluble	Moderately soluble	Moderately soluble
Gastrointestinal (GI) absorption	High	Low	Low
Bioavailability Score	0.55	0.55	0.55

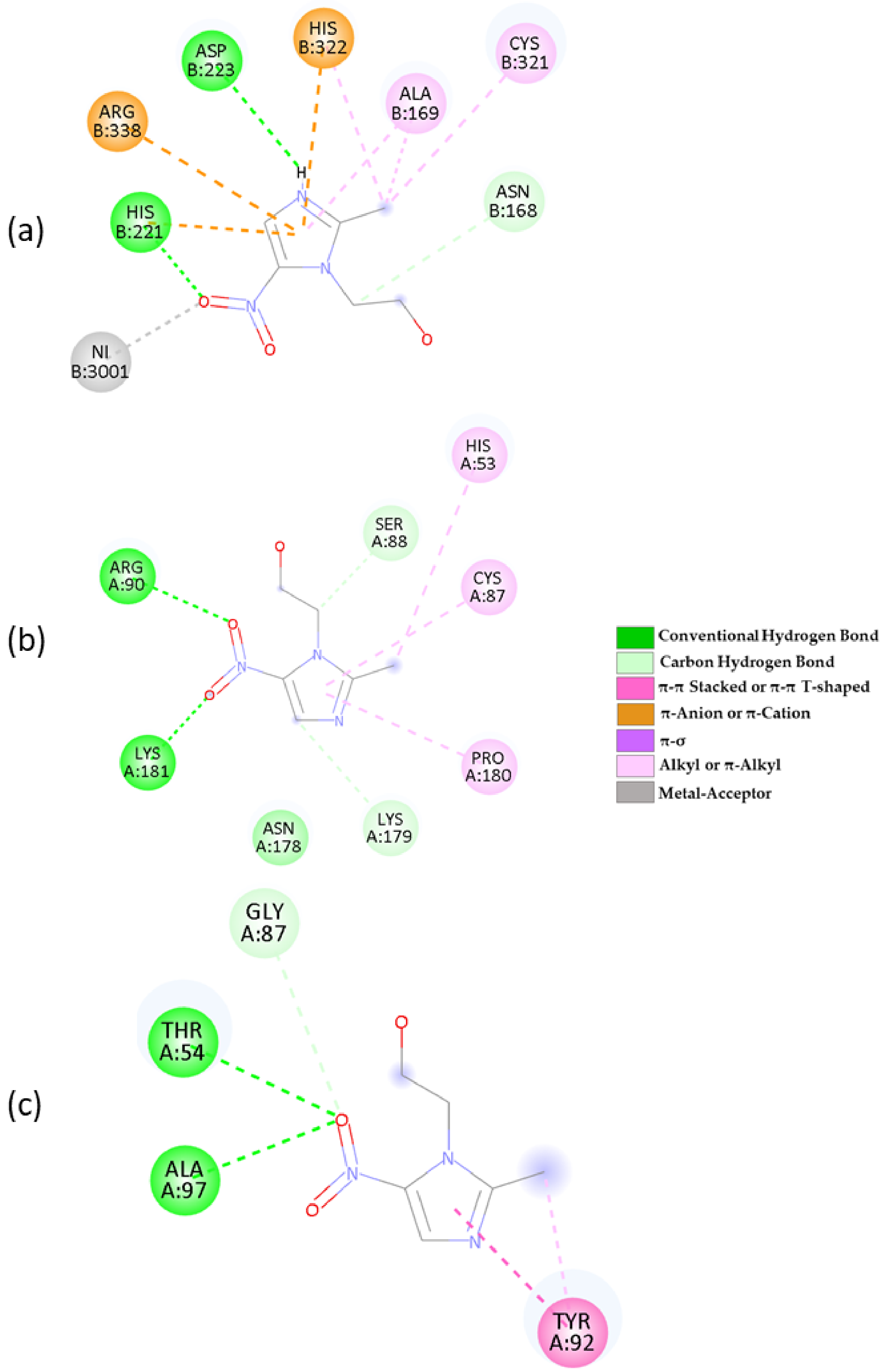

Figure S6. Two-dimensional representations for the interactions of metronidazole (**1**) with *H. pylori* targets (a) urease (1E9Y), (b) RdxA oxygen-insensitive nitroreductase (3QDL), and (c) flavodoxin (2W5U).

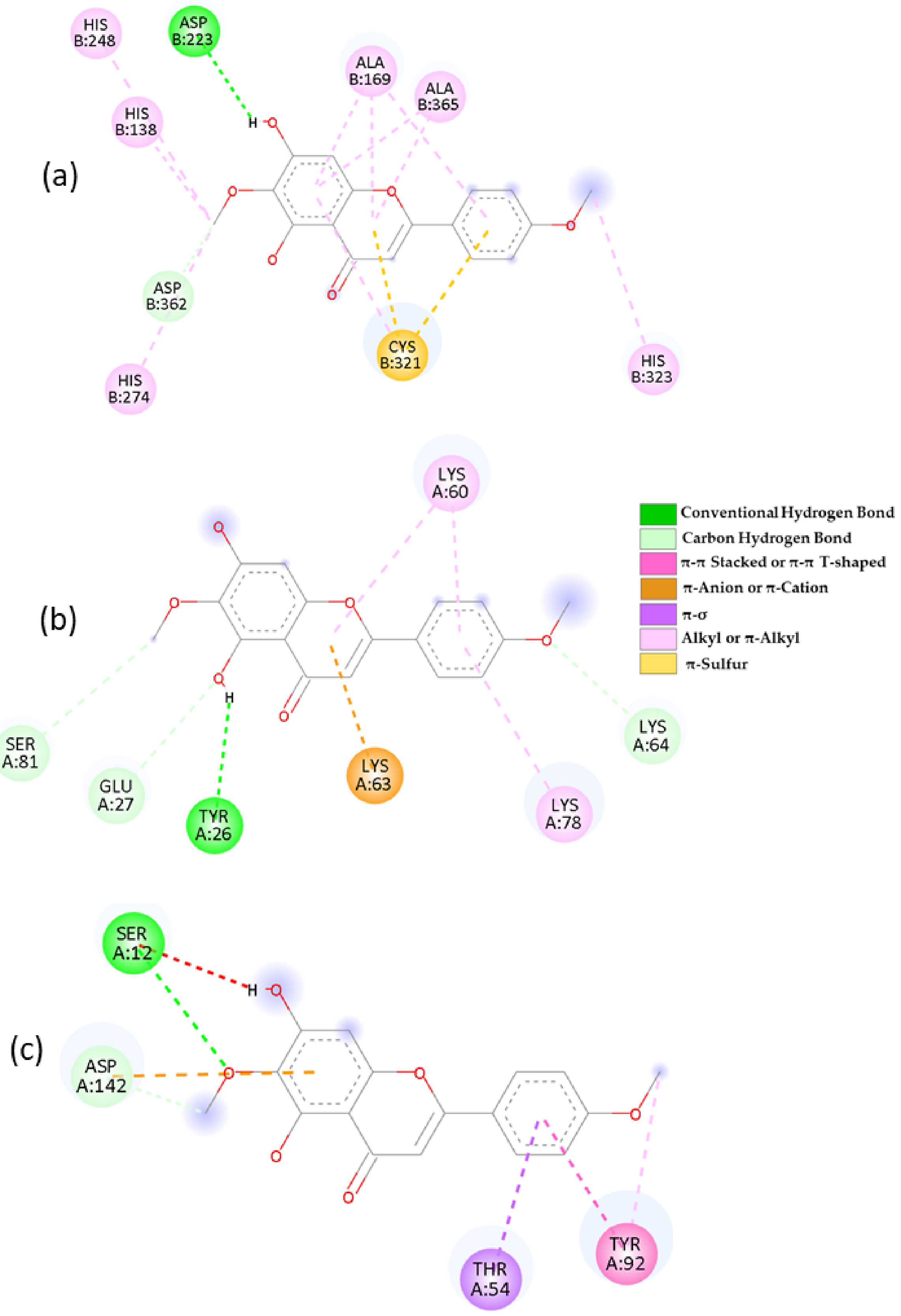

Figure S7. Two-dimensional representations for the interactions of pectolinarigenin with *H. pylori* targets (a) urease (1E9Y), (b) RdxA oxygen-insensitive nitroreductase (3QDL), and (c) flavodoxin (2W5U).

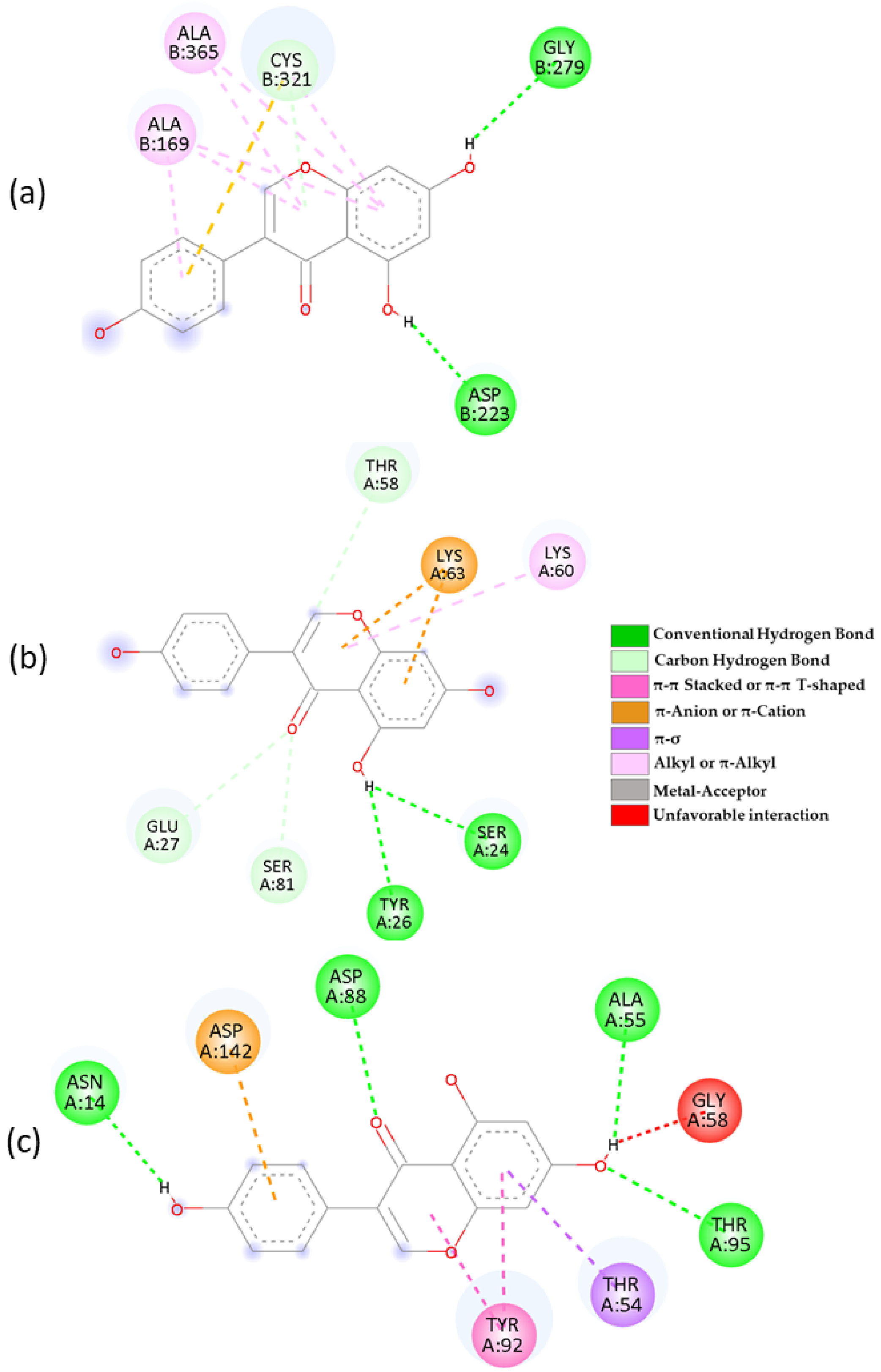

Figure S8. Two-dimensional representations for the interactions of genistein with *H. pylori* targets (a) urease (1E9Y), (b) RdxA oxygen-insensitive nitroreductase (3QDL), and (c) flavodoxin (2W5U).

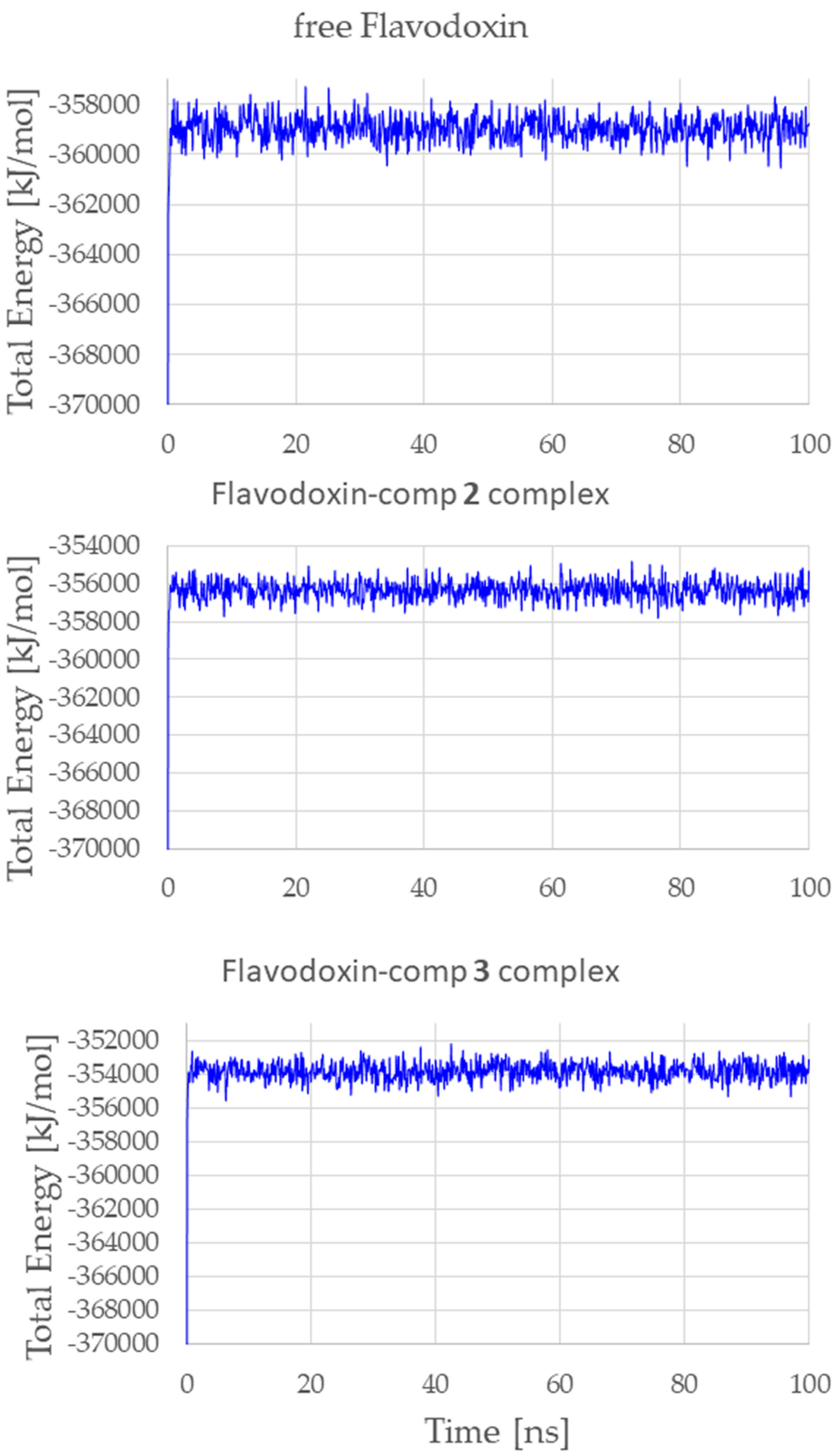

Figure S9. Data from MD simulation: total potential energy of the indicated systems during all simulation time.

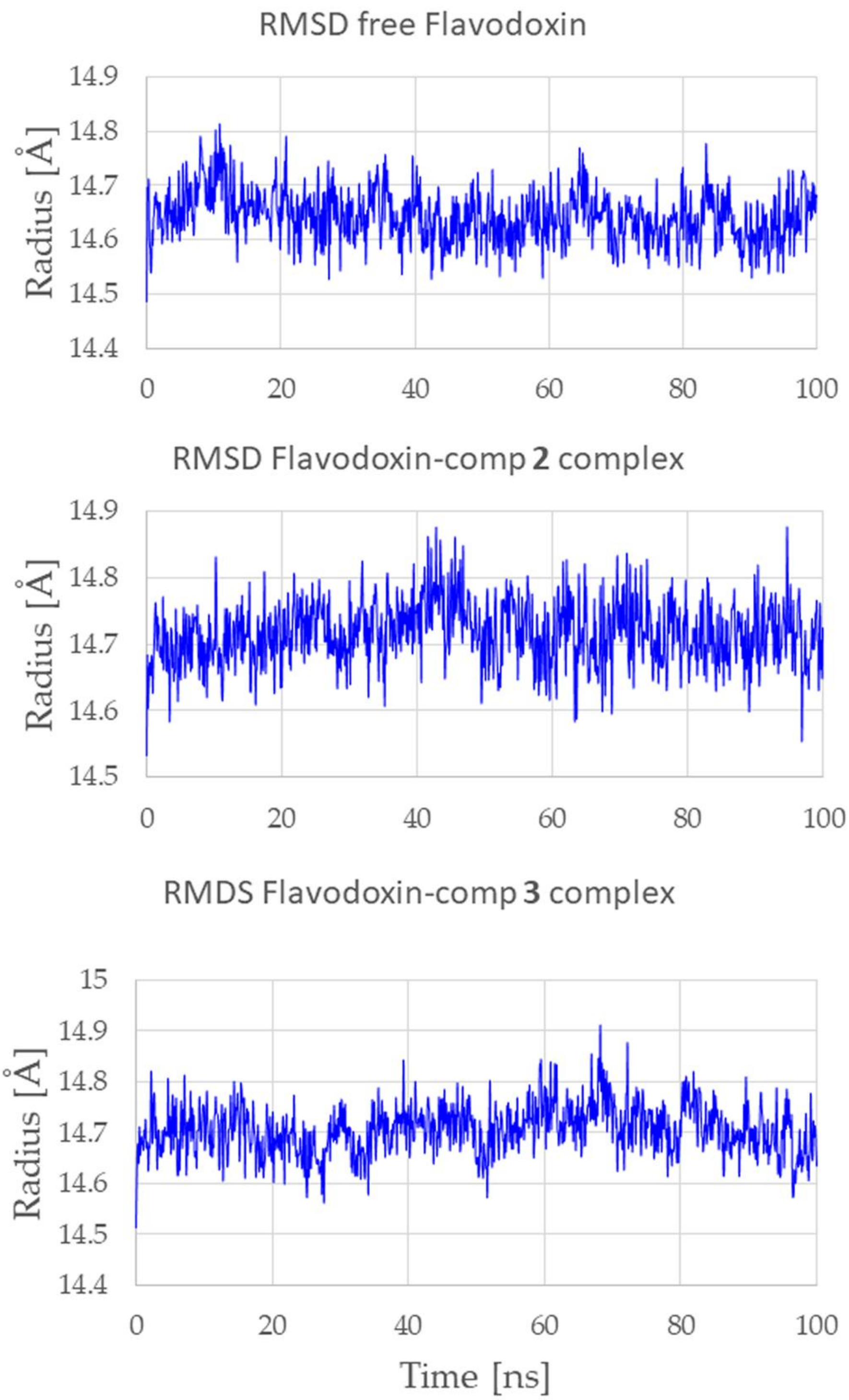

Figure S10. Data from MD simulation: radius of gyration (in Å) during all simulation time for the indicated systems.
